# Ramadan Fasting in Germany (17–18 h/Day): Effect on Cortisol and Brain-Derived Neurotrophic Factor in Association With Mood and Body Composition Parameters

**DOI:** 10.3389/fnut.2021.697920

**Published:** 2021-08-12

**Authors:** Amin Riat, Abdulhadi Suwandi, Samaneh Khoshandam Ghashang, Manuela Buettner, Luqman Eljurnazi, Guntram A. Grassl, Christoph Gutenbrunner, Boya Nugraha

**Affiliations:** ^1^Department of Rehabilitation Medicine, Hannover Medical School, Hannover, Germany; ^2^Institute of Medical Microbiology and Hospital Epidemiology, Hannover Medical School and German Center for Infection Research (DZIF), Hannover, Germany; ^3^Institute of Cell Biochemistry, Center of Biochemistry, Hannover Medical School, Hannover, Germany; ^4^Department of Dermatology, Johannes Wesling Medical Centre, Minden, Germany; ^5^Institute for Laboratory Animal Science, Hannover Medical School, Hannover, Germany

**Keywords:** intermittent fasting, ramadan, cortisol, BDNF, mood, anthropometric, body composition

## Abstract

Ramadan fasting (RF) is a type of diurnal intermittent fasting. Previous studies reported the benefits of RF in healthy subjects on mood and health related to quality of life (QoL). Cortisol and brain-derived neurotrophic factor (BDNF) have been shown to play a role in mood, body composition parameters, and health-related QoL. This study aimed at elucidating the mechanism of the benefit of RF, particularly cortisol and BNDF and their association with mood and QoL. Insulin growth factor-1 (IGF-1), interleukin (IL)-8, matrix metalloproteinase (MMP)-9, and myoglobin were determined. Thirty-four healthy men and women were recruited. Serum from peripheral venous blood samples was collected at five time points: 1 week before RF (T1); mid of RF (T2), last days of RF (T3), 1 week after RF (T4), and 1 month after RF (T5). The amounts of biological mediators in the serum samples were determined by enzyme-linked immunosorbent assay (ELISA) and Luminex assays. BDNF and cortisol significantly decreased at T3 (*p* < 0.05) and T4 (*p* < 0.001) compared to T1, respectively. It seems the benefits of RF for mood-related symptoms are mediated by different biological mediators, particularly cortisol and BDNF.

## Introduction

Fasting has been practiced for centuries not only to fulfill religious obligations, but also as therapy and lifestyle (1). Ramadan fasting (RF) is one type of diurnal intermittent fasting that is performed by millions of Muslims worldwide. RF is performed from dawn to sunset for 29–30 days. Due to geographical differences, the time periods of RF vary from 9 to 22 h per day (2). In this study, RF was performed for 17–18 h/day. During RF, food, water, smoking, and sexual activities are forbidden, but not after breaking the fast (3). Travelers, the unwell, elderly, children, and pregnant and nursing people are exempt from RF (4).

Several studies have reported benefits of RF in healthy individuals (1, 5) and patients (6–9). Benefits of fasting were reported in different patient groups, including in diabetic (6, 7, 10), asthma (11), and allergic patients (11). In healthy men and women, the benefits of RF have been shown, such as improvement of body composition parameters, mood, fatigue, and health-related quality of life (1, 5).

Since the last decade, previous studies have reported the benefits of RF and aimed to show how such improvements are mediated by various biological factors. Fasting, in general, modifies immune systems and improves the symptoms of chronic inflammatory diseases (12). It can be mediated by monocyte metabolic activity and several cytokines (12, 13). However, the results concerning inflammatory markers are still inconclusive (13–15). A previous study elucidated the effects of RF on diabetic-related parameters, such as glucometabolic markers (16). Our previous study showed no differences with regard to the level of creatinine, as a biological marker for kidney, between fasting and non-fasting groups, although RF was conducted for about 19 h (17).

Our previous studies reported the benefit of RF in health-related quality of life and mood-related symptoms (1, 5). Furthermore, its benefit could still be observed up to 1 month after finishing RF (1). Therefore, it would be of interest to elucidate the biological mechanism related to mood-related symptoms.

Cortisol is a glucocorticoid class of hormone that is produced in the adrenal cortex (18, 19). Its release occurs in a circadian rhythm based on the regulation of the hypothalamic-pituitary-adrenal axis (HPA) as well as in stressful situations and during physical work (19). High cortisol levels are associated with stress-related disorders (18, 19). During fasting, the levels of cortisol alter. Alterations differ based on measurement time (20, 21). With regard to RF, there is a lack of information on how cortisol is altered during RF, particularly when fasting is performed in the summer time for about 17 to 18 h daily. Brain-derived neurotrophic factor (BDNF) is one of the neurotrophins that has been studied in mental-related disorders (22–25), cognition (26), physical activity, and nutrition (27, 28). Furthermore, a lower level of BDNF in the peripheral nervous system is associated with impairment of cognitive performance and higher body weight (26, 29). Therefore, this study aimed to determine cortisol and BDNF levels in serum samples during RF and their association with mood and health-related quality of life.

Additionally, other biological mediators associated with nutritional intake, body composition parameters, and mood such as insulin growth factor-1 (IGF-1), myoglobin, interleukin-8 (IL-8), and matrix metalloproteinase-9 (MMP-9) were also determined. IGF-1 also plays a role in mood and body composition (30, 31). IL-8, as one of the inflammatory markers, has been known to be associated with mood and nutritional intake (32, 33). The increase of IL-8 is correlated with positive mood (33). MMP-9 is correlated with obesity (34, 35) and muscle strength (36). Meanwhile, myoglobin has been known to be responsible for intramuscular oxygen transport (37) and can be used to measure the muscle breakdown in the body (38), as our previous studies showed a decrease in skeletal muscle mass during RF (1, 5).

## Materials and Methods

All procedures were authorized in accordance with the ethical standards and approval of Hannover Medical School (Ethics No. 7242; Registration code of the trial: DRKS00017640) and the Declaration of Helsinki 1964.

### Participants

For this study, 34 healthy men and women were enrolled. Most of them were students and employees of Hannover Medical School. Inclusion criteria: healthy men and women, with no history of pain and mental health-related disorders; above the age of 18 years; intended to fast the whole month of Ramadan from sunrise to sunset. Exclusion criteria: participants who have interrupted their fasting for more than 7 days. Participation in this study was contingent on the signing of the informed consent in either English or German according to the preference of the participants.

### Study Design

**Figure 1:**
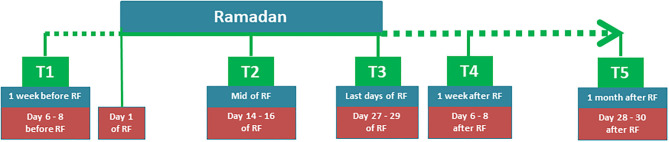
Study design.

This was an explorative study to determine the mechanism of RF based on a previous study (1). This study consisted of five time points: 1 week before RF (T1), mid of RF (T2), last days of RF (T3), 1 week after RF (T4), and 1 month after RF (T5) (see Figure 1).

### Biological Parameters

#### Blood Sampling

Peripheral venous blood samples were collected from all participants in serum tubes (Monovette®, Sarstedt, Germany) between 08:00 am to 10:00 am from T1 to T5. Serum samples were allowed to clot before centrifugation at 1,500 g (15 min) (Universal 320R, Hettich, Tuttlingen, Germany). They were stored at −80°C until analyses.

#### Detection of Biological Parameters

The blood serum concentrations of cortisol (Cortisol Pa-rameter Assay Kit, R&D Systems Inc.) was determined by employing a standard competitive enzyme-linked immunosorbent assay (ELISA) according to the manufacturer's instructions. Myoglobin and human IGF-1 serum levels were detected by sandwich ELISA using a Human Myoglobin ELISA kit (Invitrogen) and Quantikine ELISA Human IGF-1 Immunoassay (R&D Systems Inc.) according to the manufacturer's instructions. BDNF, IL-8, and MMP-9 serum levels were detected using a Human Magnetic Luminex Assay (R&D Systems), following the protocol from the manufacturer.

### Body Composition Parameters, Mood, and Health-Related Quality of Life

In this study, body composition parameters (body fat percentage (BFP), body weight (BW), body water mass (BWM), skeletal muscle mass (SMM), body fat mass (BFM) and fat-free mass (FFM), and estimated basal metabolic rate (BMR)); mood (Beck's depression inventory (BDI)-II and hospital and anxiety depression scale-Anxiety/Depression (HADS-A/D), and health-related quality of life (QoL) [fatigue: fatigue severity scale (FSS); sleepiness (Epworth sleepiness score (ESS)] were determined. The methodology and results of these parameters were reported in a previous study (1). In this article, these parameters are only reported to support the correlation with the determined biological parameters.

### Statistics Evaluation

The study objective aimed at assessing the level of cortisol, BDNF, and other biological parameters (IL-8, MMP-9, IGF-1, myoglobin) before (T1), during (T2 and T3) and after (T4 and T5) RF. The data were analyzed by using either Kruskal-Wallis or two-way Friedman ranked test. The *post hoc* tests were performed, and significances were adjusted by using Bonferroni correction. Spearman's correlation tests were performed to determine correlation between clinical parameters and biological mediators. Missing data were replaced by using a mean imputation method. The Kolmogorov–Smirnov test was used to determine the distribution of the data. Significance was set at *p* < 0.05. SPSS 26 (IBM, New York City, NY, USA) was used to analyze the data. Effect sizes were calculated by using following formula: η^2^ = Z^2^/(N - 1).

## Results

### Baseline Data

The baseline parameters of all, male, and female participants before RF (T1) are shown in Table 1. No significant differences could be seen between male and female participants in age, ethnicity, body mass index (BMI), and level of cortisol, IGF-1, MMP-9, and myoglobin. Whereas significant differences were identified in body weight (BW) and level of BDNF and IL-8.

**Table 1:** Baseline parameters (T1) in all, male, and female participants.

	**All participants *N* = 34**	**Male *N* = 19**	**Female *N* = 15**	***P* (male vs. female)**
Age	25.1 ± 0.8	24.8 ± 1.0	25.5 ± 1.2	0.65
Caucasian/other	23/11	14/5	9/6	0.48^§^
BW (kg)	72.0 ± 2.3	77.31 ± 3.1	65.3 ± 2.3	<0.01
BMI (kg/m^2^)	24.8 ± 0.6	25.10 ± 0.9	24.4 ± 0.8	0.56
Cortisol (Median(IQR))	225.5 (215.6–261.7)	229.3 (219.0–260.9)	218.7 (187.8–265.6)	0.228
BDNF (Median(IQR))	567.2 (345.7–970.9)	742.5 (516.8–1164.0)	440.4 (227.4–849.8)	0.021
IL-8 (Median(IQR))	2.5 (1.9–4.1)	3.7 (2.6–4.4)	1.9 (1.5–2.3)	0.002
IGF-1 (Median(IQR))	4579.5 (2058.5–6907.0)	4073.1 (1653.9–7514.6)	5474.0 (2999.7–6553.6)	0.784
MMP-9 (Median(IQR))	3783.3 (2463.5–7723.9)	4606.1 (3008.0–11028.2)	3177.0 (1882.0–4204.0)	0.056
Myoglobin (Median(IQR))	13.3 (8.0–21.1)	15.4 (11.2–25.5)	9.0 (6.9–15.8)	0.051

*BW, body weight; BMI, body mass index; BDNF, brain-derived neurotrophic factor; IL-8, interleukin-8; IGF-1, insulin growth factor-1; MMP-9, matrix metalloproteinase-9.*

§ *Chi square test*.

### Biological Parameters in All Participants

**Figure 2:**
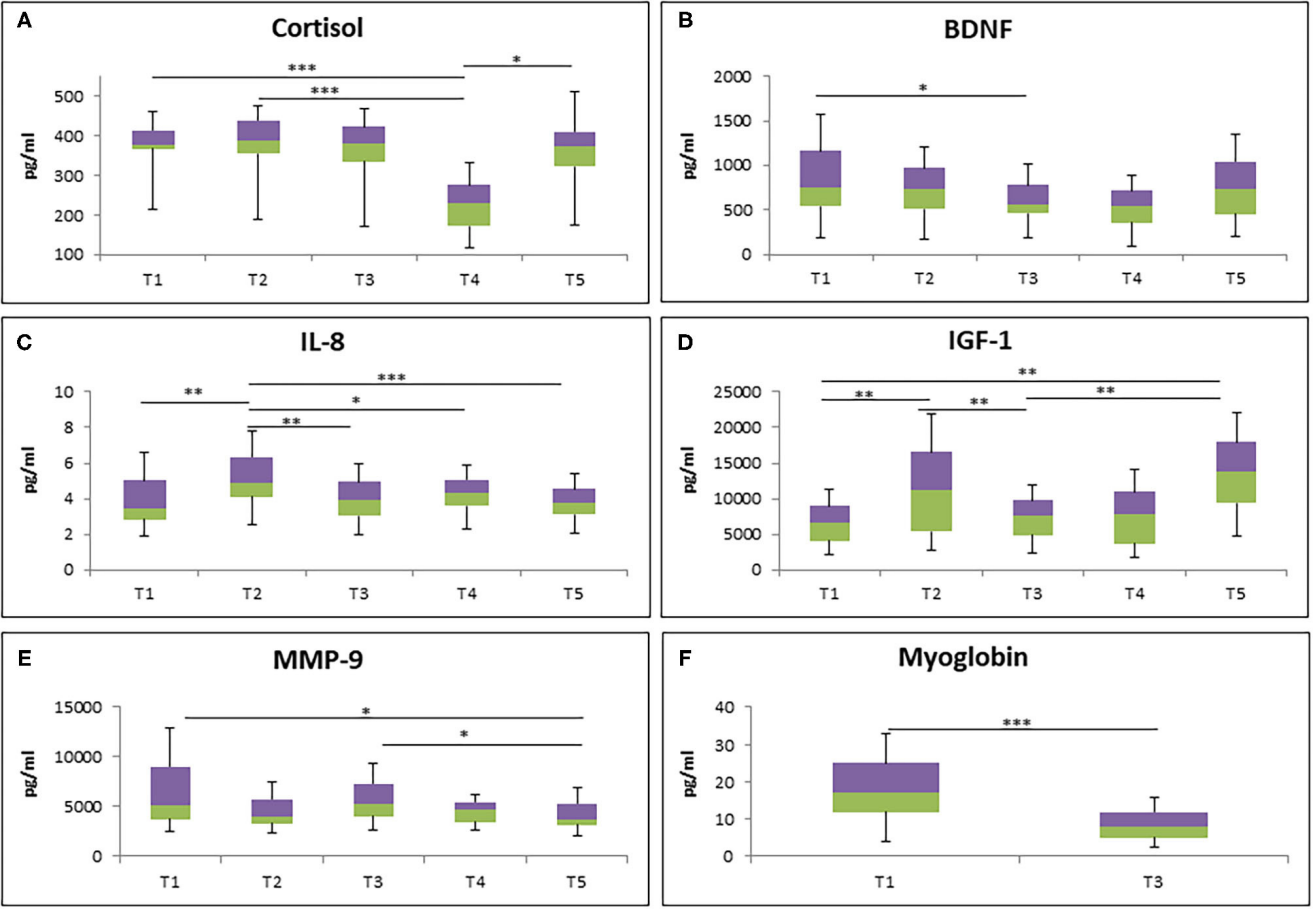
The level of cortisol **(A)**, BDNF **(B)**, IL-8 **(C)**, IGF-1 **(D)**, MMP-9 **(E)**, and myoglobin **(F)** during RF in all participants (*N* = 34). *p < 0.05, **p < 0.01, ***p < 0.001.

To study the effect of RF on biological parameters, different types of biological markers were analyzed at different time points (Figure 2). The cortisol level was significantly decreased at 1 week after RF (T4) in comparison to 1 week before RF (T1) and mid of RF (T2). However, it turned back to the baseline level 1 month after RF (T5) (Figure 2A). Next, a significantly lower level of BDNF was observed from the last days of RF (T3) to T1 (Figure 2B). Furthermore, chemokine IL-8 level was increased at T2 compared to T1, T3, T4, and T5 (Figure 2C).

IGF-1 level was also analyzed at different time points. Significantly increased level of IGF-1 at T2 compared to T1 was observed, although it was decreased at T3. Interestingly, 1 month after RF (T5), it was increased again compared to T1 and T3 (Figure 2D). Furthermore, the level of MMP9, an enzyme involved in the degradation of the extracellular matrix, was significantly decreased at T5 compared to T1 and T3 (Figure 2E). Lastly, the reduction of myoglobin level, an iron- and oxygen-binding protein in the skeletal muscle tissue, was observed at T3 compared to T1 (Figure 2F).

### Biological Parameters in Male and Female Participants

**Figure 3:**
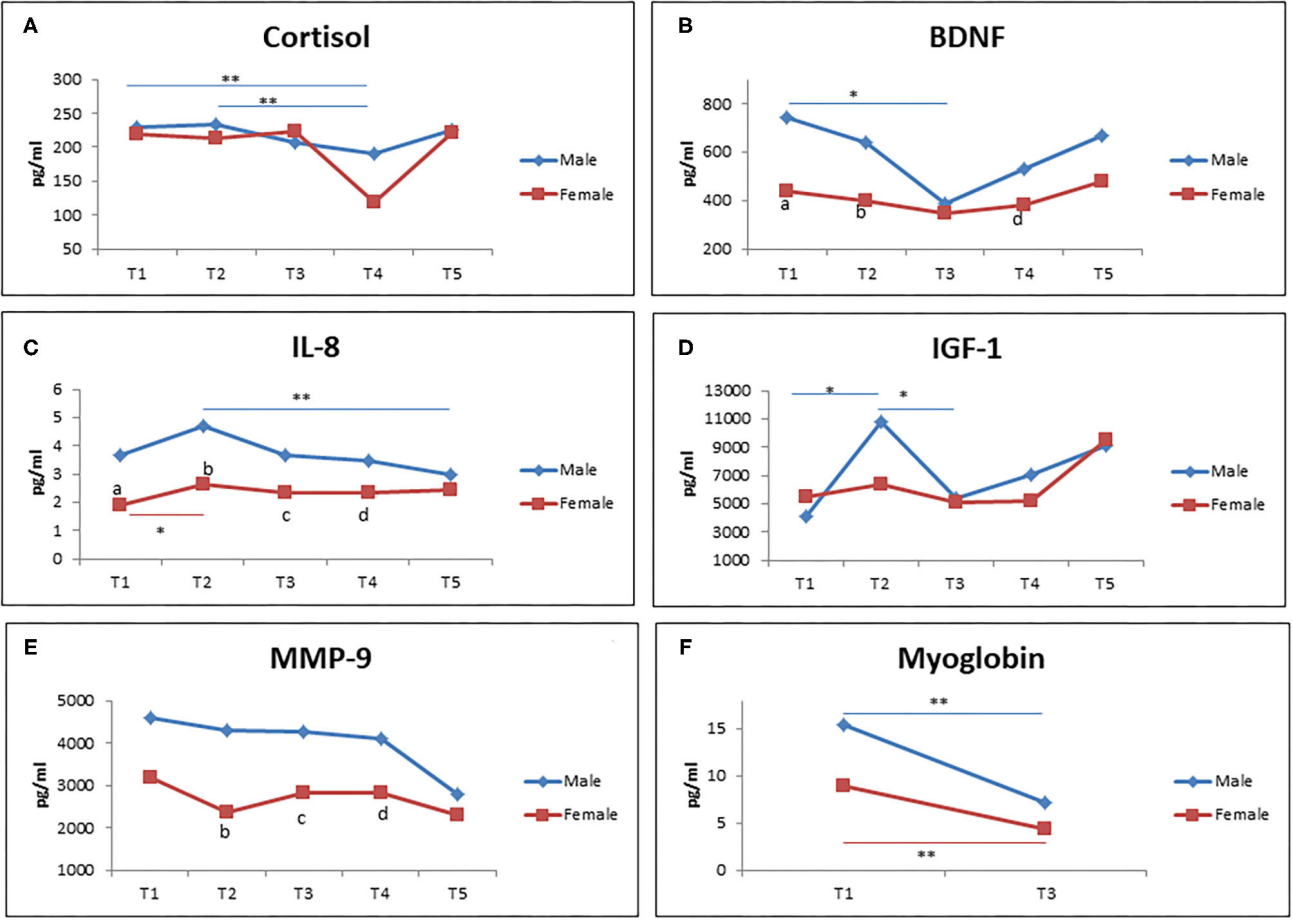
The level of cortisol **(A)**, BDNF **(B)**, IL-8 **(C)**, IGF-1 **(D)**, MMP-9 **(E)**, and myoglobin **(F)** during RF (male = 19; female = 15). **(a–e)** significant difference between men and women at T1, T2, T3, T4, and T5. *p < 0.05, **p < 0.01.

Figure 3 demonstrates different types of gender based-biological markers (men and women) during RF at different time points. Interestingly, there was a significant reduction of cortisol level from T1 to T2 in comparison to T4 in male participants, but not in female participants (Figure 3A). Similar results were observed for BDNF level in that only men showed a reduction of BDNF level from T1 to T3, but not women. In addition, male participants showed a higher level of BDNF at T1, T2, and T4 compared to female participants (Figure 3B). For IL-8 level, it was shown that it significantly increased from T1 to T2 in women. Men showed a significant decreased IL-8 level from T2 to T5. The IL-8 level was significantly lower in women in comparison with men at T1, T2, T3, and T4 (Figure 3C).

Next, we observed the increased level of IGF-1 from T1 to T2, but it came back to the baseline level at T3 in male participants. Meanwhile, no significant changes was observed in female participants (Figure 3D). Furthermore, there was no significant difference of MMP-9 levels observed at each time points in both men and women. However, the female participants showed a lower level of MMP-9 in comparison to male participants. Lastly, a significantly decreased level of myoglobin was observed from T1 to T3 in both female and male participants (Figure 3F).

### Effect Size of Biological Parameters During RF

In order to determine the magnitude of effect of RF on biological parameters at different time points, effect sizes were computed (Table 2). The calculations were performed for T1–T3; T1–T4; and T1–T5. The large effect size of cortisol was observed particularly at T1-T4 of all participants and also in both men as well as women. In men, the large effect size of cortisol could also be observed between T1 and T3. The large effect size of BDNF in all, male, and female participants was only observed at T1-T3. Its effect sizes were medium at T1–T4. The large effect size of IGF-1 and MMP-9 could be observed at T1-T5 in all, male, and female participants. However in male participants, the large effect size of IGF-1 was also observed at T1–T4. Myoglobin showed a large effect size at T1–T3 in all, male, and female participants.

**Table 2:** Effect size of biological parameters in all, male, and female participants between T1 and T3, T1 and T4, and T1 and T5.

	**Effect size (** **η^**2**^** **)**								
	**All**			**Male**			**Female**		
	**T1–T3**	**T1–T4**	**T1–T5**	**T1–T3**	**T1–T4**	**T1–T5**	**T1–T3**	**T1–T4**	**T1–T5**
Cortisol	0.061	0.592	0.015	0.15	0.660	0.067	0.005	0.516	0.001
BDNF	0.296	0.098	0.027	0.307	0.077	0.111	0.327	0.114	0.008
IL-8	0.005	0.034	0.001	0.001	0.04	0.055	0.045	0.274	0.055
IGF-1	0.000	0.124	0.415	0.011	0.164	0.468	0.018	0.100	0.331
MMP-9	0.018	0.078	0.353	0.041	0.117	0.467	0.000	0.095	0.22
Myoglobin	0.634	–	–	0.573	–	–	0.774	–	–

*Effect size (η^2^: small: 0.01–0.05; medium: 0.06–0.12; large ≥ 0.13). BDNF, brain-derived neurotrophic factor; IL-8, interleukin-8; IGF-1, insulin growth factor-1; MMP-9, matrix metalloproteinase-9*.

### Correlations Between Biological Mediators and Clinical Parameters During RF

Table 3 shows the correlations between biological mediators and clinical parameters in all, male, and female participants. At T3, in all participants, IGF-1 and IL-8 levels showed positive correlations with body composition-related parameters. Cortisol showed a negative correlation with BW. Interestingly, in men, IGF-1 levels were only positively correlated with BW, meanwhile in women, a significant negative correlation occurred between cortisol and FSS; and cortisol positively correlated with BFP. At T4, in all participants, cortisol, IL-8, and MMP-9 levels were significantly correlated with body composition-related parameters. However, only cortisol levels showed a significant negative correlation with fatigue (FSS). In male participants, IGF-1 levels were significantly correlated with body composition-related parameters. Cortisol levels were correlated with mood (BDI score) and sleepiness (ESS score) in male and female subjects, respectively. In women, cortisol was also negatively correlated with BFM and positively correlated with FFM. At T5, in all participants, cortisol level was negatively correlated with BMI and BFP. Meanwhile, MMP-9 level was negatively correlated with BFP. BDNF levels were significantly correlated with mood (HADSD score). In men, BDNF was positively correlated with BFM, BFP, and WHR. In women, BDNF levels were negatively correlated with SMM, BW, FFM, and BMR. Additionally, in women only, BDNF levels were positively correlated with mood (HADSD score). Cortisol level was also negatively correlated with BFM and BFP in women only.

**Table 3:** Correlation between biological mediators and clinical parameters in all, male, and females participants.

**Time point**	**All participants**			**Gender**	**Parameters**	**R and *P*-value**
		**Parameters**	**R and *P*-value**			
T3	IGF-1	BW BFM BMI	R:0.418;p:0.014 R:0.410; p:0.016 R:0.455; p:0.007	Male Female	IGF-1 vs. BW Cortisol vs. FSS Cortisol vs. BFP	R:0.480; p:0.038 R:−0.630;*p*: 0.012 R: −0.622; *p*:0.013
	Cortisol	BMI	R:−0.453; p:0.007			
	IL-8	SMM BWM FFM BMR	R:0.428; p:0.012 R:0.425;p:0.012 R:0.423;p:0.013 R:0.425;p:0.012			
T4	Cortisol IL-8	FSS SMM FFM BFP BW SMM Bwater FFM BMR	R:-0.358; p:0.038 R:0.345;p:0.046 0.355; p:0.040 R:-0.360;p:0.036 R:0.354;p:0.04 R:0.393;p:0.022 R:0.397;p:0.02 R:0.365;p:0.034 R:0.403;p:0018	Male Female	IGF-1 vs. BW IGF-1 vs. BFM IGF-1 vs. BMI IGF-1 vs. WHR Cortisol vs. BDI Cortisol vs. BFM Cortisol vs. ESS Cortisol vs. FFM	R:0.545;p:0.016 R:0.566;p:0.011 R:0.463;p:0.046 R:0.531;p:0.019 R:-0.555; p:0.014 R:-0.534;p:0.04 R:-0.516; p:0.049 R:0.543; p:0.037
	MMP-9	SMM Bwater FFM BMR	R:0.446;p:0.008 R:0.454,p:0.007 R:0.458;p:0.006 R:0.455; p:0.007			
T5	Cortisol	BMI BFP	R:-0.471;p:0.005 R:-0.377;p:0.028	Male	BDNF vs. BFM BDNF vs. BFP BDNF vs. WHR	R:0.512,p:0.025 R:0.561; p: 0.012 R: 0.551; p:0.015
	BDNF MMP-9	HADSD BFP	R:0.438;p:0.010 R:-0.342;p:0.048	Female	Cortisol vs. BFM Cortisol vs. BFP BDNF vs. SMM BDNF vs. BW BDNF vs. FFM BDNF vs. BMR BDNF vs. HADSD	R:-0.586; p:0.022 R:-0.650; p:0.009 R:-0.597; p:0.019 R:-0.604; p:0.017 R:-0.599; p:0.018 R:-0.600; p:0.018 R:0.624; p:0.013

*BDNF, brain-derived neurotrophic factor; IL-8, interleukin-8; IGF-1, insulin growth factor-1; MMP-9, matrix metalloproteinase-9; BW, body weight; BMI, body mass index; BFM, body fat mass; SMM, skeletal muscle mass; FFM, fat free mass; BMR, basal metabolic rate; BFP, body fat percentage; ESS, Epworth Sleepiness Scale; HADSD, Hospital Anxiety Depression Scale-Depression; BDI, Beck's Depression Scale-II; FSS, fatigue severity score*.

## Discussion

This study aimed to particularly determine cortisol and BDNF levels and their correlation with mood, health-related quality of life, and body composition parameters during RF. Additionally, other biological mediators including IGF-1, IL-8, MMP-9, and myoglobin were also studied. The RF was performed for 17–18 h/day for 1 month.

### Cortisol

Cortisol has been known to be associated with mental health-related problems (18, 30, 39, 40) and regulated by circadian rhythms (30). In healthy subjects, cortisol also plays a role in regard to physical activity, nutrition changes, and mood symptoms (24, 31). In this study, the level of cortisol decreased, particularly at T3 and T4 in all participants. However, the decrease at both time points could only be observed in male participants. It was confirmed by the correlation of cortisol with mood (BDI) at T4 in male participants. This study is in agreement with Bahijri et al. who showed the decrease of cortisol level after RF (32), but contradicts that of Al Rawi et al. (33). The difference could be explained by the fact that the latter study determined cortisol level in overweight and obese participants, whereas in this current study, the BMI of participants was in the normal range (1). In this study, the difference of cortisol level only occurred in male participants. This could be attributed to the fact that men and women manage stress differently (34). This result is in agreement with the mood level of men that has been reported in previous studies (1, 5). Interestingly, cortisol level is also significantly correlated with body composition parameters. These results are supported by several other studies that showed that cortisol played a role in body composition parameters (30, 39, 40).

### BDNF

BDNF is the second neurotrophin that has been mostly determined in association with mood (17), stress-related disorders (35), nutrition (28), and chronic pain (22–24). RF has been reported to be associated with mood and alteration of nutritional intake. In this study, BDNF level of all participants tended to decrease until the last day of RF (T3) and it returned to baseline value (T1) at 1 month after RF (T5). The difference was particularly observed in male participants when compared at T1 and T3. This result cannot be directly compared with another study that did not show significant alterations, due to differing experimental time points (31); the latter study only observed BDNF level at day 3 of an intermittent fasting period. In another study, the result of the BDNF level was conflicting at T2 and T3 (17). It seems that gender could play a role in regard to this difference (36). Other reasons may include differences in body composition (1) and hormonal status (36). A significant correlation of BDNF with body composition parameters and mood supports the role of BDNF in mediating RF-derived health-related benefits and improvements (24, 27, 28).

### IL-8

IL-8 is an inflammatory mediator, which is produced in different types of tissue (37). IL-8 has also been known to be associated with mood and nutritional intake (38, 41). In this study, the level of IL-8, particularly compared to T2, was gradually decreasing until T5. This result is different from other studies (14, 15). In one study, there was no significant alteration of IL-8 (14). Meanwhile, in another study, significant alterations were observed (15). However, in the latter study, if we check at a specific time point (morning time: 0600), there was no significant difference. The discrepancies of results with the latter study (15) could be that sleep/wake pattern, meal composition, and energy expenditure were controlled. In this study, we did not control such parameters, as we preferred to observe free-living participants. In this study, significant changes of IL-8 occurred only when comparing T2 and T5; and T1 and T2 in male and female participants, respectively. Interestingly, IL-8 was significantly correlated with body composition-related parameters. This study was in agreement with other study by Razmjou et al. (42), but contradicted another study by Ghashang et al. that did not show any correlation with body composition-related parameters (14). The reason could be that the latter study only included male participants.

### IGF-1

IGF-1 is an important hormone, particularly in regulation of the pituitary growth hormone (43, 44). IGF-1 also plays a role in different clinical-related issues, such as mood and body composition (45, 46). In our study, the level of IGF-1 increased at T2 which then stabilized until 1 week after RF if we compare with before RF (T1). These data did not support other studies that showed a decrease of IGF-1 (47, 48). The differences could be the characteristics of the participants: one study observed the effect of RF on IGF-1 in overweight and obese participants, and another study only observed male participants. In our study, we had both male and female participants with normal BMI (1). These two studies only reported in two time points. Meanwhile, in our study, the measurements were performed at five different time points. In this study, the correlation of IGF-1 and body composition parameters were particularly observed at T3 (all participants and men) and T4 (in only men). This is in agreement with other studies that reported a correlation between IGF-1 and body composition parameters (46). These results support the role of IGF-1 in mediating RF-derived improvements in regard to body composition parameters (46).

### MMP-9

MMP-9 is involved in different types of pathological remodeling including inflammatory mechanism, cardiovascular disease (49), and oncologic processes (50). It is known that fasting is recommended as a complementary therapy for cancer patients (51). In this study, the decrease of MMP-9 was observed until T5. Unfortunately, there was no comparison study available in regard to MMP-9, except one that measured MMP-9 from the ocular surface (52). Furthermore, it seems the benefit of fasting in different diseases could be mediated by the decrease of MMP-9. As in different type of diseases, MMP-9 was higher (e.g., in colorectal cancer, ulcerative colitis, and inflammatory bowel diseases) (50). In this study, correlation between MMP-9 and body composition parameters occurred, particularly with BFP and SMM. This interesting finding was supported by a previous study that reported a negative correlation between MMP-9 and obesity (53, 54) and muscle strength (55).

### Myoglobin

Myoglobin is a muscle protein that is located in the cytoplasm of the heart and skeletal muscles and is responsible for intramuscular oxygen transport (56). Some studies showed that myoglobin can be used to measure the muscle breakdown in the body (57). Our previous studies showed a decrease of skeletal muscle mass (1, 5). Therefore, in this study, myoglobin was determined. Myoglobin was determined only at T1 (one week before RF) and T3 (last days of RF) as it was the most interesting time point that might lead to the highest effect during RF in regard to the decrease of SMM (1, 5). Interestingly, the level of myoglobin significantly decreased from T1 to T3. However, there was no correlation between myoglobin and SMM at all time points. It seems the decrease of myoglobin at T3 was due to the decreased level of SMM in the body. Therefore, the body would need less myoglobin. However, further studies are needed for this to be elucidated and other studies should also be performed in different time points.

### Effect Size

The effect sizes of these biological parameters were determined to observe the magnitude alterations due to RF. Based on effect size calculations, it seems all determined biological parameters played a role in bringing benefits of RF on body composition parameters, mood, and QoL, as almost all parameters showed large effect sizes, either at T3, T4, and T5. These results were in line with the effect size of RF on mood and body composition parameters in our previous study (1).

## Limitation

This study has some limitations. As mentioned above, all of these parameters were influenced not only by fasting, but also physical activities and nutrition intake. In this study, we also did not record the number of participants who skipped predawn breakfast and their sleeping pattern, which could also influence all the parameters. Therefore, further studies need to consider recording these parameters. This study was participated by young participants (mostly students in the Hannover area), which could influence the level of cortisol and other biological parameters. Future studies should be considered to include participants with a broader range of ages. This study was only performed in the fasting group. Future studies to compare fasting groups and non-fasting groups would also be of interest.

## Strengths

Some strong points of this study include the determination of RF in both male and female participants. Many RF studies only reported pre/during and post RF (9, 48, 58). However, this study determined the effect of RF in the mid of RF (T2); and extended the determination until 1 week (T4) and 1 month after RF (T5), in order to determine the longer effect of RF. Many RF studies have been conducted particularly at equatorial, subequatorial, and tropical zones which mostly perform RF between 13 to 16 h/day every year (9, 13, 33, 47, 58, 59). This study was performed in the northern part of the globe (Germany—temperate zones). In this zone, during summer, RF is performed longer than those aforementioned zones.

## Implications of the Findings and Future Directions

The results of this study show that a prolonged duration of fasting (17 to 18 h/day) could still be tolerated and even show benefits for both physical and mental health in young and healthy participants. The benefits might be mediated by different biological mediators. Religious fasting, particularly RF, has been a concern of both patients and health professionals. Therefore, future studies with similar duration (17–18 h/day or even longer) of fasting should also be performed in different health conditions in order to give the best recommendations for patients who live in the temperate zone.

## Conclusions

The benefits of RF include improvement of mood and body composition-related parameters. The benefit of RF in mood and body composition-related parameters might be mediated by different biological mediators, particularly cortisol and BDNF.

## Data Availability Statement

The datasets used and/or analyzed during the present study are available from the corresponding author on reasonable request.

## Ethics Statement

The studies involving human participants were reviewed and approved by Ethics Committee of Hannover Medical School (Ethics No. 7242; Registration code of the trial: DRKS00017640). The patients/participants provided their written informed consent to participate in this study.

## Author Contributions

BN: conceptualization, formal analysis, visualization, and supervision. BN, AS, and MB: methodology and software. BN and AS: validation. Investigation: BN, AR, LE, SG, AS, and MB. Resources and funding acquisition: CG and GG. Data curation: BN, AR, LE, AS, and MB. Writing—original draft preparation: BN, AR, and SG. Writing—review and editing: BN, AS, SG, LE, MB, CG, and GG. Project administration: AR and LE. All authors have read and agreed to the published version of the manuscript.

## Conflict of Interest

The authors declare that the research was conducted in the absence of any commercial or financial relationships that could be construed as a potential conflict of interest.

## Publisher's Note

All claims expressed in this article are solely those of the authors and do not necessarily represent those of their affiliated organizations, or those of the publisher, the editors and the reviewers. Any product that may be evaluated in this article, or claim that may be made by its manufacturer, is not guaranteed or endorsed by the publisher.
